# Recurrent pancreatic cancer treated with N-803 and PD-L1 t-haNK followed by an EGFR-targeted nanocell drug conjugate

**DOI:** 10.1093/oncolo/oyae267

**Published:** 2024-10-04

**Authors:** Katayoun Moini, Tara Seery, Chaitali Nangia, Jennifer MacDiarmid, Himanshu Brahmbhatt, Patricia Spilman, Lennie Sender, Patrick Soon-Shiong

**Affiliations:** Chan Soon-Shiong Institute for Medicine (CSSIFM), El Segundo, CA 90245, United States; Chan Soon-Shiong Institute for Medicine (CSSIFM), El Segundo, CA 90245, United States; Chan Soon-Shiong Institute for Medicine (CSSIFM), El Segundo, CA 90245, United States; EnGeneIC, Ltd., North Ryde, Sydney, NSW 2113, Australia; EnGeneIC, Ltd., North Ryde, Sydney, NSW 2113, Australia; ImmunityBio, Inc., Culver City, CA 90232, United States; ImmunityBio, Inc., Culver City, CA 90232, United States; ImmunityBio, Inc., Culver City, CA 90232, United States

**Keywords:** metastatic pancreatic cancer, PD-L1 t-haNK, N-803, E-EDV-D682, EDV-α-galactosyl ceramide

## Abstract

Multimodal temporal therapy orchestrated to leverage immunotherapy, tumor-targeted chemotherapy, and natural killer (NK) cell therapy may provide an opportunity to induce immunogenic cell death for tumor response and increased survival in patients with recurrent cancer. The interleukin-15 (IL-15) superagonist N-803, an enhancer of NK cells, CD4 + T cells, cytotoxic CD8 + T cells, and memory T-cell activity, combined with off-the-shelf PD-L1-targeted high-affinity NK (PD-L1 t-haNK) cells represent novel immunotherapies designed to overcome an immunosuppressive tumor microenvironment (TME). The epidermal growth factor receptor-targeted antibody-nanocell conjugate E-EDV-D682 provides tumor-targeted chemotherapy in the form of its anthracycline metabolite PNU159682 (nemorubicin) cargo and is currently being studied in combination with immunomodulatory EDVs delivering the adjuvant α-galactosyl ceramide (GC). Here, we report the compassionate use treatment of this combination in a patient with recurrent, metastatic pancreatic cancer (mPC) after 3 lines of therapy. Under the initial single-patient Investigational New Drug (spIND) protocol, the patient received N-803, PD-L1 t-haNK cells, and the albumin doxorubicin conjugate aldoxorubicin for ~27 months. The patient’s disease became stable on this regimen, and a transient complete response was observed by ~14 months of therapy. Due to progression, a second spIND protocol was designed whereby the patient received E-EDV-D682 plus EDV-GC for more than 24 months, which resulted in stable disease and the patient’s continued survival at the time this report was written. The patient’s extended survival despite the dire prognosis associated with recurrent mPC points to the merits of this temporal combination regimen in overcoming immuno-chemo resistance with enhanced immune activity required for tumor response and extended survival.

## Teaser

This article presents a case report for a patient who had received 3 lines of therapy for recurrent metastatic pancreatic cancer and who then underwent compassionate-use, single-patient Investigational New Drug treatment, initially with a combination of N-803, PD-L1-t-haNK, and aldoxorubicin; followed by ongoing treatment with E-EDV-D682 plus EDV-GC.

**Implications for practice**

A patient with recurrent, metastatic pancreatic cancer after 3 lines of therapy achieved stable disease and then a transient complete response to compassionate-use treatment with the IL-15 superagonist N-803 combined with PD-L1-targeted high-affinity NK cells and the albumin doxorubicin conjugate aldoxorubicin. Subsequent compassionate use of the EGFR-targeted antibody-nanocell conjugate E-EDV-D682 resulted in stable disease and the patient’s continued survival. The patient’s extended survival despite her dire prognosis points to the merits of these temporal combination therapeutic regimens in overcoming immuno-chemo resistance with the enhanced immune activity required for tumor response and extended survival.

## Introduction

Pancreatic cancer (PC) is the 4th leading cause of cancer-related death worldwide and typically has a poor prognosis, with a 5-year survival rate of 5%, and a life expectancy of ~3-6 months for patients with metastatic pancreatic cancer (mPC).^1^

The National Comprehensive Cancer Network (NCCN) recommends FOLFIRINOX or nab-paclitaxel (Abraxane) plus gemcitabine as 1st-line therapy and gemcitabine mono- or combination therapy as 2nd-line therapy. Options for additional therapy are capecitabine or fluoropyrimidine plus oxaliplatin.^2-4^ There is no standard of care (SoC) for 3rd-line therapy, although modest benefit has been reported for tegafur-gimeracil-oteracil potassium or nano-liposomal irinotecan plus 5-FU/leucovorin.^5,6^

Options for patients with pancreatic cancer who have persistent disease after 3 lines of therapy are few, and palliative care is often the only choice.

While immunotherapy has been found to be effective in the treatment of many types of cancer, PC is generally resistant to checkpoint inhibitor monotherapy likely due to impairments in T-cell function and an immunosuppressive tumor microenvironment (TME).^7-10^

The investigational biologic N-803, an Fc-conjugated interleukin-15 superagonist, may overcome therapeutic resistance. N-803 has been shown to enhance natural killer (NK), CD4 + T, and CD8 + killer T-cell activation, leading to the generation of memory T cells and durable complete responses when used in combination with other therapies.^11-13^ Examples of N-803 efficacy in combination therapy include the achievement of durable complete responses in all patients in a phase 1b trial of N-803 plus bacillus Calmette-Guerin (BCG) in BCG-naïve non-muscle invasive bladder cancer (NMIBC)^14^ and in a phase 2/3 trial of the combination in BCG-unresponsive NMIBC.^15^ Based findings in the latter study, the US FDA recently approved the combination for the treatment of bladder carcinoma in situ with or without Ta/T1 papillary disease.^15,16^

To further circumvent immunosuppression, N-803 may be used in combination with investigational programmed death receptor ligand 1-targeted high-affinity natural killer (PD-L1 t-haNK) cells which have demonstrated activity against immunosuppressive myeloid-derived suppressor cells (MDSCs),^17,18^ and/or the investigational chemotherapy aldoxorubicin—doxorubicin with an acid-sensitive linker—that has enhanced activity at the tumor site.^19^ Investigational vaccines targeting tumor-associated antigens (TAAs) such as carcinogenic embryonic antigen or CEA (ETBX-051) and/or mucin-1 or MUC1 (ETBX-061) may also enhance responses by inducing antigen-specific T cells targeting tumor cells.^20,21^

While the investigational agents N-803, aldoxorubicin, PD-L1-t-haNK, ETBX-051, and -061 have not yet been approved by the US FDA for use in pancreatic cancer, they have been studied in multiple phase I/II QUILT (Quantum Integrative Lifelong Trial) clinical studies to explore safety and early signals of efficacy across multiple tumor types, including pancreatic cancer.^22-25^ To date, over 300 subjects have participated in the QUILT trials wherein the combination immunotherapy was well-tolerated with evidence of complete responses in late-stage tumors (BCG-unresponsive non-muscle invasive bladder, triple-negative breast, pancreatic, and head/neck cancer, as well as Merkel cell carcinoma),^26,27^ supporting Institutional Review Board (IRB) approvals for their use in the present case.

An additional therapeutic approach leverages both the advantages of tumor-targeting and the stimulation of an immune response. The novel Antibody Nanocell Drug Conjugate (ANDC) E-EDV-D682 packages PNU159682, the potent secondary metabolite of the anthracycline nemorubicin,^28^ and delivers it via the epidermal growth factor receptor (EGFR)-targeted nanocellular vector E-EDV. EDVs (EnGeneIC Dream Vectors) are bacterially-derived nanocells that can deliver cargos such as chemo- or immuno-therapies selectively to the tumor due to the tumor-associated leaky vasculature.^29-31^ To further increase efficacy, EDVs carrying an immunomodulatory adjuvant α-galactosyl ceramide cargo (EDV-GC) may be used in combination with E-EDV-D68.^32^

E-EDV-D682 plus EDV-GC therapy is being studied in clinical trials for mPC and has been demonstrated to be well-tolerated with no dose-limiting toxicities (DLTs), and no drug-related severe adverse event (SAEs); further, early efficacy signals suggest the ability of the drug combination to elicit partial responses (PR) or stable disease (SD) in patients who have exhausted treatment options.^29,33^ These findings informed the approval by the IRB for the use of E-EDV-D682/GC in the present case.

The sources of the investigational agents, their mechanism of action, and roles in therapy are listed in Table 1.

**Table 1.** Investigational* agent description, mechanisms of action, and role in treatment

Agent	Description	Source	Mechanism(s) of action	Role						
				Tumor cell cytotoxicity	Release of TAAs	Coordinate ICD signals	Mitigate TME immuno-suppression	Enhance immune responses	Condition dendritic and T cells	Maintain immune responses
**PDL-1 t-haNK**	Programmed death receptor ligand 1 targeted high-affinity natural killer cells	ImmunityBio, Inc.	Increases NK cell anti-tumor function					✓		✓
**N-803**	Nogapendekin alfa inbakicept interleukin-15 superagonist	ImmunityBio, Inc.	Increase NK and T-cell proliferation and activation					✓	✓	✓
**Aldoxorubicin**	Acid-sensitive linker conjugated doxorubicin	ImmunityBio, Inc.	Inhibits DNA repair, generates cytotoxic free radical	✓	✓	✓	✓			
**ETBX-051, 061**	Adenovirus-vectored tumor antigen vaccines; 051- brachyury, 061 - MUC1	Etubics**	Leads to killing of tumor cells that express brachyury or MUC1						✓	
**E-EDV-D682**	Epidermal growth factor receptor (EGFR)(V)-EnGeneIC Delivery Vehicle (EDV) nanovesicle-delivered PNU-159682	EnGeneIC***	Anthracycline, inhibits DNA synthesis	✓	✓	✓	✓	✓		
**EDV-GC**	EDV-delivered α-galactosyl ceramide	EnGeneIC***	Adjuvant immune enhancer				✓	✓		✓

^*^Investigational agents are not yet approved for the target indication (here, pancreatic cancer); **Etubics is wholly owned by ImmunityBio, Inc.; ***EnGeneIC EDV products licensed by ImmunityBio, Inc..

Abbreviations: 5-FU, 5-fluorouracil; ICD, Immunogenic cell death; TAAs, tumor-associated antigens.

TME, Tumor microenvironment

## Case report

Herein, we present a case report for a patient who had received 3 lines of therapy for recurrent mPC and who then underwent compassionate-use, single-patient Investigational New Drug (spIND) treatment initially with a combination of N-803, PD-L1-t-haNK, and aldoxorubicin; followed by ongoing treatment with E-EDV-D682 plus EDV-GC. The patient was provided spIND care at the Chan Soon-Shiong Institute for Medicine (CSSIFM) in El Segundo, California in partnership with Hoag Hospital.

The patient, who at the time of this report shows no signs of disease progression, is a 65-year-old female originally diagnosed with locally-advanced PC approximately 5 years ago. She had reported abdominal pain and magnetic resonance cholangiopancreatography (MRCP) revealed a 3.2 cm mass at the head of the pancreas with dilatation of the common bile duct (CBD); CT of the abdomen and pelvis revealed a 4.1 × 3.3 cm mass with associated biliary dilatation. Endoscopic ultrasound-guided fine-needle aspiration (EUS-FNA) × 3 suggested a neuroendocrine tumor (later determined by pathology to be pancreatic adenocarcinoma) and enlarged lymph nodes were noted. In the same month as her diagnosis, endoscopic retrograde CP (ERCP) was performed with placement of a CBD stent, and pathology confirmed PC. Soon after, she suffered cholangitis and cholecystitis, and underwent CT-guided placement of a cholecystostomy tube.

STRATA genome sequencing of the biopsy characterized the tissue as microsatellite stable (MSS), Tumor Mutational Burden (TMB) low, PD-LI-low, and STRATA immune signature low.^34^ No genomic alterations were detected.

As 1st-line therapy, she received 8 cycles of neo-adjuvant FOLFIRINOX over 4 months starting 1 month after diagnosis. During that time, there was an occasion when she was admitted for ascending cholangitis.

As 2nd-line therapy, she received radiation with capecitabine (Xeloda) in 28 treatments over a month starting 1 month after completion of FOLFIRINOX therapy.

Three months later, she was scheduled for planned surgical resection of the primary tumor (Whipple procedure), but diagnostic laparoscopy demonstrated metastatic disease in the liver that did not appear on CT or PET scans. A biopsy of the liver metastasis confirmed mPC and the surgery was aborted.

As 3rd-line therapy, she began treatment with gemcitabine and nab-paclitaxel immediately after the confirmation of mPC. A PET CT 2 months later showed increased uptake in the pancreatic head, suggesting a lack of response to therapy.

### PD-L1 t-haNK, N-803, and aldoxorubicin spIND therapy

Having exhausted 3 lines of SoC therapy, a compassionate-use treatment plan was designed and approved for this patient comprising the immune-cell activating interleukin-15 superagonist N-803, allogenic PD-L1-targeted high-affinity natural killer cells (PD-L1 t-haNK) and the linker-conjugated doxorubicin compound, aldoxorubicin. This investigational therapy was alternated with gemcitabine plus nab-paclitaxel.

**Figure 1.**
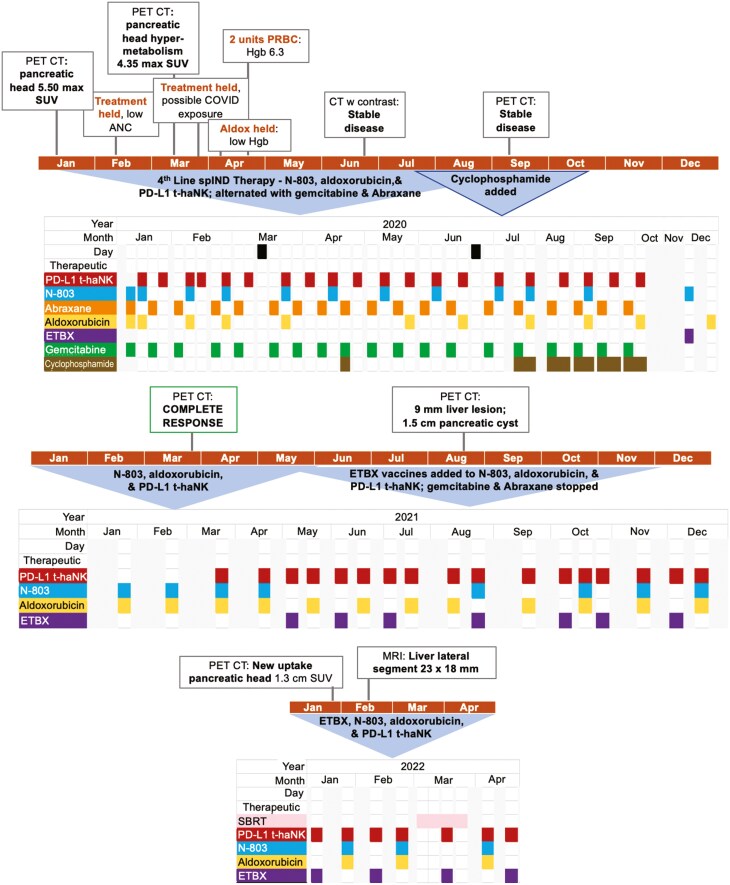
*Initial spIND therapy with N-803, aldoxorubicin, and PD-L1 t-haNK*. The patient received the initial compassionate-use therapy as shown by year. Any adverse events, blood chemistry or other findings that affected treatment are shown in comment boxes above the timeline.

As shown in Figure 1, 4th-line spIND therapy began within weeks of the noted lack of response to previous therapy, following a schedule of alternating therapies for ~9 months. Treatment was held in month 2 due to low ANC and in month 3 due to possible coronavirus disease exposure. Aldoxorubicin only was held and 2 units of PRBCs were given in month 4 due to low hemaglobin. A dose of cyclophosphamide was given at 3 months, and regular administration of cyclophosphamide 6-9 months after initiation of spIND therapy.

CT and PET CT were performed at months 5 and 8 of spIND therapy showed stable disease.

Eleven months after the initiation of spIND therapy, the investigational human adenovirus serotype 5 (hAd5) vector-based cancer vaccines ETBX-051 (targeting carcinogenic embryonic antigen; CEA) and -061 (targeting mucin-1; MUC1) were added, and treatment with gemcitabine/nab-paclitaxel ceased.

Notably, after ~14 months of spIND therapy, no abnormal metabolic activity was observed on PET CT, suggestive of a complete response (CR) (Figure 1). Subsequent PET CTs performed 19 and 24 months (2 years) after commencement of spIND therapy revealed the presence of a new lateral segment liver lesion likely representing progressive disease. At that time the patient required routine paracentesis due to abdominal ascites. The initial spIND regimen was continued for a total of 2 years and 3 months. In the final month, the patient received radiation therapy to the liver.

### E-EDV-D682 plus EDV-GC spIND therapy

Given the patient’s progression after a complete response to the initial spIND therapy, a new compassionate-use treatment plan based on a recently approved Investigational New Drug (IND) application for pancreatic ductal adenocarcinoma (PDAC) of the antibody nanocell drug conjugate packaging nemorubicin, E-EDV-D682, plus the immunomodulatory adjuvant EDV-α-galactosyl ceramide, EDV-GC, was initiated.

**Figure 2.**
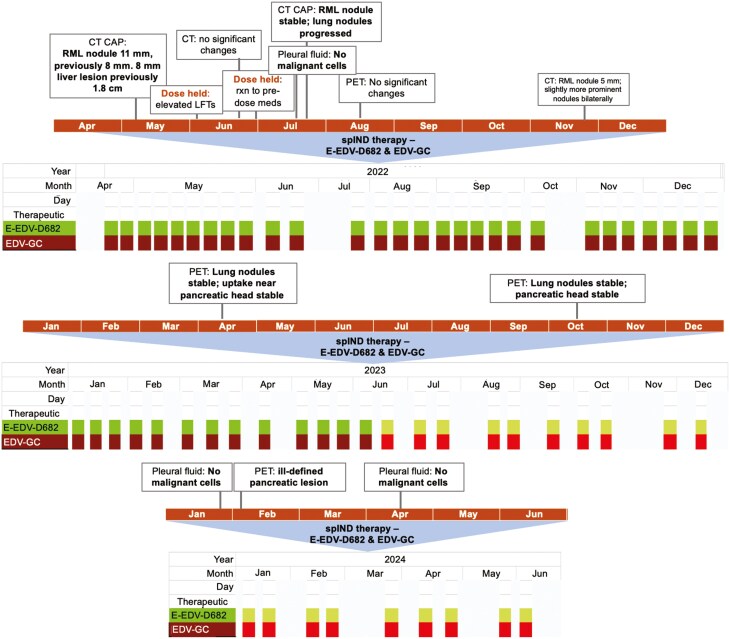
*spIND therapy with E-EDV-D682 and EDV-GC.* The patient received a second compassionate use therapy regimen as shown by year. The initial dosing regimen comprising two repeat doses is shown in darker colors and the single dosing regimen (started after approximately 14 months of therapy) in lighter colors. Any adverse events, blood chemistry, or other findings that affected treatment are shown in comment boxes above the timeline. The dosing regimen was ongoing as of June 2024.

As shown in Figure 2, immediately after cessation of the first spIND regimen, 2 sequential doses of E-EDV-D682 plus EDV-GC were administered bi-weekly for 4 weeks (16 doses). On the treatment day, the 1st dose was followed 45 (+ 15) minutes after completion by a 2nd repeat dose. In month 2 of this regimen, a dose was held due to elevated liver function tests (LFTs), and one was held later that month due to the patient’s reaction to pre-dose medications.

Subsequent cycles consisted of a maintenance regimen of 2 repeat doses of E-EDV-D682 plus EDV-GC delivered 45 (+ 15) minutes apart starting 10 months from initiation of the second spIND regimen, followed by a regimen of a single dose of E-EDV-D682 plus EDV-GC commencing 4 months after that; for both regimens, dosing was every 2 weeks.

Noting that the patient’s disease is characterized by being diffuse and not clearly defined on imaging, findings from imaging and the absence of malignant cells in pleural fluid collected frequently throughout treatment confirm a lack of disease progression and are suggestive of a response to therapy for at least an additional 12 months, at which time this report was written.

## Discussion

Herein we describe the case of a patient with advanced, recurrent metastatic pancreatic cancer who—after exhausting 3 lines of standard therapy—achieved a complete response with the combination of N-803, PD-L1 t-haNK, aldoxorubicin, and a single dose of CEA- and MUC-based adeno (Ad5) vaccines. Following the patient’s progression, she then began a new spIND regimen comprising investigational E-EDV-D682 plus adjuvant EDV-GC therapy which prevented disease progression and supported the patient’s continued survival.

Standard chemotherapies—FOLFIRINOX, capecitabine, and gemcitabine—as well as radiation all have tumor cell cytotoxicity and thus elicit release of tumor-associated antigens from dying cells that coordinate immunogenic cell death (ICD) signaling.^35^ In an immunosuppressive TME, however, the magnitude of ICD needed to elicit a response to therapy is unlikely. Nab-paclitaxel offers an opportunity to mitigate TME immunosuppression by its albumin-bound form,^36,37^ but is not always sufficient.

N-803, PD-L1 t-haNK, aldoxorubicin, and the ETBX TAA-targeted vaccines all address the problem of immunosuppression by different mechanisms: N-803 by direct activation of NK and T cells,^38,39^ PD-L1 t-haNK by the provision of an exogenous source of NK cells that not only can assist in overcoming endogenous NK insufficiency but also the expression of immunosuppressive PD-L1 and, importantly, the presence of MDSCs.^17,18^

Aldoxorubicin, in a fashion similar to nab-paclitaxel, allows for a greater concentration of effective chemotherapy at the tumor site, with a reduction of off-target effects that can limit the dose or frequency of dosing with other chemotherapies.^19^

The vaccines have the potential to then add to responses by directing the immune system to surviving tumor cells expressing TAAs and thus conditioning dendritic and T cells to recognize these tumor cells if they recur.

The response of this patient to this first spIND regimen was not completely unexpected, as some advanced PC patients in QUILT clinical trials have been observed to respond to similar treatment regimens,^22,24^ but is nonetheless notable due to the patient’s treatment history and prognosis.

The subsequent response to additional spIND therapy is also explained by the mechanisms of action of E-EDV-D682 and EDV-GC. E-EDV-D682 is targeted directly to cancer cells via an anti-EGFR antibody. Its bilayer membrane protects the drug payload, facilitating the use of the highly cytotoxic compound PNU159682, which has the potential to overcome drug resistance. The E-EDV-D682 particle size prevents extravasation into non-target tissues, allowing delivery of higher concentrations of effective therapy to the targeted tumor with no payload-related off-target effects^28^; efficacy is increased by co-delivery of the immuno-enhancer EDV-GC, with similarly protected cargo.^29^ In this patient, E-EDV-D682 plus EDV-GC therapy has prevented disease progression and prolonged survival with minimal toxicities. This is in alignment with findings from a clinical trial of E-EDV-D682/GC in patients with advanced pancreatic cancer wherein partial response (PR) or stable disease (SD) was achieved at 8 weeks in 8/9 patients.^29,33^

The EDV-based treatment regimen also benefited the patient by reducing the need for long infusion times. Currently, available treatments for mPC are highly toxic and time-intensive, often requiring patients to endure hours of infusions. For this patient, E-EDV-D682 offered a novel, targeted approach to the delivery of effective chemotherapy, with minimal toxicities and a significant reduction in infusion time, ultimately contributing to improved quality of life and resumption of regular activities.

The prolonged survival and tumor response of this patient with advanced PC provides encouraging validation that temporal/spatial orchestration of the innate and adaptive immune systems to elicit immunogenic cell death raises the possibility of converting metastatic cancer to a chronic disease. Further evidence of the efficacy of this approach is provided by the durable complete responses observed in patients with BCG-naïve and BCG-unresponsive non-muscle invasive bladder cancer.^14,15^ While N-803 (ANKTIVA) plus BCG has been approved for the treatment of BCG-unresponsive NMIBC, the investigational therapies described herein are currently only available for patients with pancreatic cancer through a compassionate use or participation in a clinical trial.

## Data Availability

All data are presented in the manuscript or are available upon reasonable request.

## References

[1] Carrato A , Falcone A, Ducreux M, et al. A systematic review of the burden of pancreatic cancer in Europe: real-world impact on survival, quality of life and costs. J Gastrointest Cancer. 2015;46:201-211. 10.1007/s12029-015-9724-1 25972062 PMC4519613

[2] Tempero MA , Malafa MP, Al-Hawary M, et al. Pancreatic adenocarcinoma, version 2.2021, NCCN Clinical Practice Guidelines in Oncology. J Natl Compr Canc Netw. 2021;19:439-457. 10.6004/jnccn.2021.0017 33845462

[3] Suker M , Beumer BR, Sadot E, et al. FOLFIRINOX for locally advanced pancreatic cancer: a systematic review and patient-level meta-analysis. Lancet Oncol. 2016;17:801-810. 10.1016/S1470-2045(16)00172-8 27160474 PMC5527756

[4] Miki M , Fujimori N, Ueda K, et al. Treatment effect and safety of nanoliposomal irinotecan with fluorouracil and folinic acid after gemcitabine-based therapy in patients with advanced pancreatic cancer: a multicenter, prospective observational study. J Clin Med. 2022;11:5084. 10.3390/jcm11175084 36079012 PMC9457338

[5] Kim B , Ahn J, Jung JH, et al. Efficacy of the third-line chemotherapy in patients with advanced pancreatic cancer. J Clin Oncol. 2023;41:711-711. 10.1200/jco.2023.41.4_suppl.711

[6] Luo D , Liao S, Li Q, et al. Case report: a case of locally advanced pancreatic cancer which achieved progression free for over 12 months by subsequent therapy with anlotinib hydrochloride plus tegafur-gimeracil-oteracil potassium (TS-1). Front Oncol. 2022;12:862600. 10.3389/fonc.2022.862600 35847852 PMC9283868

[7] Bear AS , Vonderheide RH, O’Hara MH. Challenges and opportunities for pancreatic cancer immunotherapy. Cancer Cell. 2020;38:788-802. 10.1016/j.ccell.2020.08.004 32946773 PMC7738380

[8] Christenson ES , Jaffee E, Azad NS. Current and emerging therapies for patients with advanced pancreatic ductal adenocarcinoma: a bright future. Lancet Oncol. 2020;21:e135-e145. 10.1016/S1470-2045(19)30795-8 32135117 PMC8011058

[9] Roth MT , Cardin DB, Berlin JD. Recent advances in the treatment of pancreatic cancer. F1000Res. 2020;9:F1000 Faculty Rev-131. 10.12688/f1000research.21981.1

[10] Schizas D , Charalampakis N, Kole C, et al. Immunotherapy for pancreatic cancer: a 2020 update. Cancer Treat Rev. 2020;86:102016. 10.1016/j.ctrv.2020.102016 32247999

[11] Wrangle JM , Velcheti V, Patel MR, et al. ALT-803, an IL-15 superagonist, in combination with nivolumab in patients with metastatic non-small cell lung cancer: a non-randomised, open-label, phase 1b trial. Lancet Oncol. 2018;19:694-704. 10.1016/S1470-2045(18)30148-7 29628312 PMC6089612

[12] Acoba JD , Rock A, Wong HC. Phase Ib/II study of ALT-803 in combination with gemcitabine and nab-paclitaxel in patients with advanced pancreatic cancer. J Clin Oncol. 2017;35:TPS510-TPS510. 10.1200/jco.2017.35.4_suppl.tps510

[13] Margolin K , Morishima C, Velcheti V, et al. Phase I trial of ALT-803, a novel recombinant IL15 complex, in patients with advanced solid tumors. Clin Cancer Res. 2018;24:5552-5561. 10.1158/1078-0432.CCR-18-0945 30045932 PMC6239933

[14] Rosser CJ , Tikhonenkov S, Nix JW, et al. Safety, tolerability, and long-term clinical outcomes of an IL-15 analogue (N-803) admixed with bacillus calmette-guérin (BCG) for the treatment of bladder cancer. Oncoimmunology. 2021;10:1-7. 10.1080/2162402X.2021.1912885 PMC8096327 33996264

[15] Chamie K , Chang Sam S, Kramolowsky E, et al. IL-15 superagonist NAI in BCG-unresponsive non–muscle-invasive bladder cancer. NEJM Evidence. 2022;2:1-11. 10.1056/EVIDoa2200167 38320011

[16] FDA. FDA approves nogapendekin alfa inbakicept-pmln for BCG-unresponsive non-muscle invasive bladder cancer. US Food and Drug Administration. Accessed May 12, 2024; https://www.fda.gov/drugs/resources-information-approved-drugs/fda-approves-nogapendekin-alfa-inbakicept-pmln-bcg-unresponsive-non-muscle-invasive-bladder-cancer

[17] Fabian KP , Padget MR, Donahue RN, et al. PD-L1 targeting high-affinity NK (t-haNK) cells induce direct antitumor effects and target suppressive MDSC populations. J ImmunoTher Cancer. 2020;8:e000450. 10.1136/jitc-2019-000450 32439799 PMC7247398

[18] Lee MY , Robbins Y, Sievers C, et al. Chimeric antigen receptor engineered NK cellular immunotherapy overcomes the selection of T-cell escape variant cancer cells. J ImmunoTher Cancer. 2021;9:e002128. 10.1136/jitc-2020-002128 33741731 PMC7986659

[19] Gong J , Yan J, Forscher C, Hendifar A. Aldoxorubicin: a tumor-targeted doxorubicin conjugate for relapsed or refractory soft tissue sarcomas. Drug Des Devel Ther. 2018;12:777-786. 10.2147/DDDT.S140638 PMC5896668 29670334

[20] Gatti-Mays ME , Redman JM, Donahue RN, et al. A phase I trial using a multitargeted recombinant adenovirus 5 (CEA/MUC1/Brachyury)-based immunotherapy vaccine regimen in patients with advanced cancer. Oncologist. 2019;25:479-e899. 10.1634/theoncologist.2019-0608 31594913 PMC7288633

[21] Gabitzsch ES , Tsang KY, Palena C, et al. The generation and analyses of a novel combination of recombinant adenovirus vaccines targeting three tumor antigens as an immunotherapeutic. Oncotarget. 2015;6:31344-31359. 10.18632/oncotarget.5181 26374823 PMC4741610

[22] Seery T , Lee J, Sender L, et al. NANT cancer vaccine an orchestration of immunogenic cell death by overcoming immune suppression and activating NK and T cell therapy in patients with third line or greater metastatic pancreatic cancer. J Clin Oncol. 2019;37:TPS4-TP63.

[23] Seery T , Nangia C, McKean H, Sender L, Reddy S, Soon-Shiong P. QUILT-88: NANT pancreatic cancer vaccine in 3rd, 4th, and 5th line advanced disease. ASCO GI Meeting 2022, San Francisco, CA.

[24] Seery TE , Kistler M, Nangia CS, et al. Immunotherapy combining NK and T cell activation with IL-15 super agonist (N-803), off-the-shelf high-affinity CD16 NK (haNK) or PDL1 targeted haNK and checkpoint inhibitor in relapsed/refractory advanced pancreatic cancer. J Clin Oncol. 2020;38:e15015-e15015. 10.1200/jco.2020.38.15_suppl.e15015

[25] Seery TE , Nangia CS, McKean HA, et al. Phase 2 Quilt 88 trial of DAMP inducers combined with IL15 superagonist, N-803, and anti–PD-L1 NK cell therapy more than doubles historical overall survival in patients with third- to sixth-line advanced pancreatic cancer. J Clin Oncol. 2022;40:4147-4147. 10.1200/jco.2022.40.16_suppl.4147

[26] Drusbosky L , Nangia C, Nguyen A, et al. Complete response to avelumab and IL-15 superagonist N-803 with Abraxane in Merkel cell carcinoma: a case study. J ImmunoTher Cancer. 2020;8:e001098. 10.1136/jitc-2020-001098 32913030 PMC7484858

[27] Kistler M , Soon-Shiong P, Lee J, Nangia C, Sender L, Jones F. NANT Cancer Vaccine an orchestration of immunogenic cell death by overcoming immune suppression and activating NK and T cell therapy in patients with third line or greater TNBC and head & neck SCC. SITC 2018, Washington, D.C.; P310.

[28] Holte D , Lyssikatos JP, Valdiosera AM, et al. Evaluation of PNU-159682 antibody drug conjugates (ADCs). Bioorg Med Chem Lett. 2020;30:127640. 10.1016/j.bmcl.2020.127640 33127540

[29] Lundy J , Marx GM, MacDiarmid J, Brahmbhatt H, Ganju V. Interim data: Phase I/IIa study of EGFR-targeted EDV nanocells carrying cytotoxic drug PNU-159682 (E-EDV-D682) with immunomodulatory adjuvant EDVs carrying α-galactosyl ceramide (EDV-GC) in patients with recurrent, metastatic pancreatic cancer. J Clin Oncol. 2020;38:4632-4632. 10.1200/jco.2020.38.15_suppl.4632

[30] MacDiarmid JA , Brahmbhatt H. Minicells: versatile vectors for targeted drug or si/shRNA cancer therapy. Curr Opin Biotechnol. 2011;22:909-916. 10.1016/j.copbio.2011.04.008 21550793

[31] MacDiarmid JA , Mugridge NB, Weiss JC, et al. Bacterially derived 400 nm particles for encapsulation and cancer cell targeting of chemotherapeutics. Cancer Cell. 2007;11:431-445. 17482133 10.1016/j.ccr.2007.03.012

[32] Sagnella SM , Yang L, Stubbs GE, et al. Cyto-immuno-therapy for cancer: a pathway elicited by tumor-targeted, cytotoxic drug-packaged bacterially derived nanocells. Cancer Cell. 2020;37:354-370.e7. 10.1016/j.ccell.2020.02.001 32183951

[33] Ganju V , Marx G, Pattison S, et al. Phase I/IIa trial in advanced pancreatic ductal adenocarcinoma treated with cytotoxic drug-packaged, EGFR-targeted nanocells and glycolipid-packaged nanocells. Clin Cancer Res. 2024;30:304-314. 10.1158/1078-0432.CCR-23-1821 37976042

[34] Tomlins SA , Khazanov NA, Bulen BJ, et al. Development and validation of an integrative pan-solid tumor predictor of PD-1/PD-L1 blockade benefit. Commun Med. 2023;3:14. 10.1038/s43856-023-00243-7 36750617 PMC9905474

[35] Troitskaya OS , Novak DD, Richter VA, Koval OA. Immunogenic cell death in cancer therapy. Acta Naturae. 2022;14:40-53. 10.32607/actanaturae.11523 35441043 PMC9013441

[36] Chen N , Li Y, Ye Y, et al. Pharmacokinetics and pharmacodynamics of nab-paclitaxel in patients with solid tumors: disposition kinetics and pharmacology distinct from solvent-based paclitaxel. J Clin Pharmacol. 2014;54:1097-1107. 10.1002/jcph.304 24719309 PMC4302229

[37] Springfeld C , Jäger D, Büchler MW, et al. Chemotherapy for pancreatic cancer. Presse Med. 2019;48:e159-e174. 10.1016/j.lpm.2019.02.025 30879894

[38] Felices M , Chu S, Kodal B, et al. IL-15 super-agonist (ALT-803) enhances natural killer (NK) cell function against ovarian cancer. Gynecol Oncol. 2017;145:453-461. 10.1016/j.ygyno.2017.02.028 28236454 PMC5447472

[39] Kim PS , Kwilas AR, Xu W, et al. IL-15 superagonist/IL-15RαSushi-Fc fusion complex (IL-15SA/IL-15RαSu-Fc; ALT-803) markedly enhances specific subpopulations of NK and memory CD8+ T cells, and mediates potent anti-tumor activity against murine breast and colon carcinomas. Oncotarget. 2016;7:16130-16145. 10.18632/oncotarget.7470 26910920 PMC4941302

